# Ethical Conflicts Experienced by Nurses in Geriatric Hospitals in South Korea: “If You Can’t Stand the Heat, Get Out of the Kitchen”

**DOI:** 10.3390/ijerph17124442

**Published:** 2020-06-20

**Authors:** Moonok Kim, Younjae Oh, Byunghye Kong

**Affiliations:** 1 Department of Nursing, Donggang University, Dongmun-Daero 50, Gwangju 61200, Korea; gaea513@hanmail.net; 2 College of Nursing, Research Institute of Nursing Science, Hallym University, Hallymdaehakgil 1, Chuncheon, Gangwon-do 24252, Korea; 3 Department of Nursing, College of Medicine, Chosun University, Pilmun-Daero 309, Dong-Gu, Gwangju 61452, Korea; bhgong@chosun.ac.kr

**Keywords:** ethical conflicts, phenomenological study, geriatric hospital, nurses

## Abstract

Ethical conflicts among nurses can undermine nurses’ psychological comfort and compromise the quality of patient care. In the last decade, several empirical studies on the phenomena related to ethical conflicts, such as ethical dilemmas, issues, problems, difficulties, or challenges, have been reported; however, they have not always deeply explored the meaning of ethical conflicts experienced by nurses in geriatric care. This study aims to understand the lived experiences of ethical conflict of nurses in geriatric hospitals in South Korea. A phenomenological study was conducted. In-depth, face-to-face interviews were performed with nine registered nurses who cared for elderly patients in geriatric hospitals in South Korea between August 2015 and January 2016. Three main themes emerged from the analysis: (1) confusing values for good nursing, (2) distress resulting from not taking required action despite knowing about a problem, and (3) avoiding ethical conflicts as a last resort. It was found that for geriatric nurses to cope with ethical conflicts successfully, clear ethical guidance, continuing ethics education to improve ethical knowledge and moral behaviors, and a supportive system or program to resolve ethical conflicts involving nurses should be established.

## 1. Introduction

Nurses commonly encounter ethical situations that are not always addressed adequately and thereby find themselves in a state of ethical conflict. Ethical conflict is often referred to as moral conflict and it is used as an interchangeable term in the nursing literature [1,2,3]. Moral conflict is defined as “any situation where normative factors such as moral principles or values clash and require incompatible actions” [4]. Experiencing ethical conflicts can give rise to several types of psychological discomfort for nurses, such as disappointment, helplessness, anger, or outrage, which could worsen difficulties in dealing with ethical conflicts [5,6]; consequently, they can impact the quality of patient care [7].

In geriatric care settings, nurses engage in more continuous and complex ethical situations related to the unique needs and vulnerability of the elderly [8], ageism [9], end-of-life care [10], or utilizing advances of technology relating to life and death [11] than nurses in any other care settings. Providing nursing care for elderly patients is replete with ethical issues because nurses deliberate about value-loaded caring and best practices concerning health, illness, life, and death [12]. Nursing goals are established to promote elderly patients’ physical, emotional, and social needs while at the same time ensuring that the patients are treated with dignity and respect [13,14]. It is no surprise that nurses in geriatric hospitals facing diverse ethical situations in their everyday practice could experience ethical conflicts.

Furthermore, the number of elderly people with chronic illness has been increasing rapidly worldwide [15], and long-term care facilities in some countries have reported a shortage of nursing staff as a main concern [2,16]. This imbalance between demand and supply is connected with a decrease in the quality of patient care and an increase in ethical conflicts in nursing. As in other countries, demand for long-term care is rapidly increasing in South Korea due to an ageing society [17]. Although Koreans traditionally used to care for their parents by living together at home, over half of Koreans currently influenced by Western individualism prefer their parents to stay in nursing homes [18]. Accordingly, the number of geriatric hospitals increased rapidly from 988 in 2011 to 1560 in 2018 [19]. Geriatric hospitals in South Korea have a legal minimum requirement regarding nurse staffing of 1 registered nurse to 6 patients, and to 2.5 patients in general hospitals. Moreover, less than two-thirds of registered nurses can be replaced with certified nursing assistants [20]. In considerations of this, care quality can no longer be guaranteed, and this inevitably exacerbates ethical conflicts involving nurses in geriatric hospitals in South Korea.

In previous literature on ethical conflicts involving geriatric nurses, the phenomenon of ethical conflicts has been examined using terms such as ethical issues [2], dilemmas [12,21], problems [22], challenges [14], barriers [23], and moral distress [24,25]. Most of these studies placed more emphasis on the sources of such phenomena. Ethical conflicts have emerged from incompatible goals related to care or outcomes among patients, surrogates, nurses, physicians, or organizations. For instance, nurses have perceived that inadequate health care they disagreed with was one of the barriers or problems for ethical nursing in long-term care facilities [22,23]. Moral distress among nurses was mainly caused by futile or inadequate care or treatments contrary to patient wishes [24,25]. In particular, inadequate care due to lack of resources or incompetent healthcare providers was the most frequently perceived ethical conflict by nurses who desired to provide best practices for elderly patients [2,14,24].

To resolve such ethical conflicts, other healthcare professionals’ willingness and support to discuss nurses’ conflicts have been useful, although few studies have investigated this topic [14,25]. One quantitative study described that nurses who reported a lack of debate on ethical problems faced higher emotional exhaustion [24]. In addition to positive dialogues, nurses believed that knowledge of ethics among staff can contribute to the better handling of ethical challenges in geriatric care settings [14]. Nursing scholars have also emphasized that nurses need to reflect on whether their ethical principles are sufficiently helpful in practicing good care for the elderly [12,26]. For ethical principles, an ethics of care approach has been suggested rather than medical ethics, called principlism and consisting of respect for autonomy, beneficence, maleficence, and justice because this may enable nurses to be more attentive and responsive to the patients’ perspective [12,26].

Although the general characteristics and tendencies of ethical conflicts by geriatric nurses have been revealed in previous studies, little is known about personal unique experiences on ethical conflicts and the essence of ethical conflicts as experienced by nurses in geriatric hospitals, which was our main research question. It is crucial to listen to nurses’ experiences of ethical conflicts through their real voices to acquire knowledge for encouraging and supporting them in resolving their ethical conflicts practically.

A phenomenological approach enables us to understand the meaning of a personal unique experience in depth by using “bracketing” of past knowledge and attitudes of phenomenological reduction [27,28,29,30]. Bracketing refers to the process of identifying and holding in abeyance preconceived beliefs and opinions about the phenomenon under study [27]. Attitudes of phenomenological reduction mean researchers put aside their worldly influences to discover the essential meaning of the phenomenon [29]. Even though neither process can ever be totally achieved, researchers bracket out the world and any presuppositions, to the extent possible, so as to confront the data in their pure form [27]. Through these phenomenological methods, researchers can discover essential structures of phenomena as lived within everyday lives [30]. Thus, the purpose of this study was to conduct an exploration of the meaning of ethical conflicts experienced by nurses in geriatric hospitals from their perspectives using a phenomenological approach.

## 2. Methods

### 2.1. Design

This phenomenological study aimed to understand and then describe the nature of ethical conflicts experienced by the nurses in geriatric hospitals by using in-depth interviews. All of the interviews started with the following question based on the principles of phenomenology [27,29]: “How do you experience ethical conflicts?” To deepen the understanding of the participants’ experiences, follow-up questions were asked: “Would you describe this experience in more detail?” and “What did this experience mean to you?”

The study was conducted in accordance with the Declaration of Helsinki and was approved by the Institutional Review Board of the Chosun University before data collection (IRB NO. 2015-0030). Written informed consent concerning the study purpose, including guaranteed anonymity and confidentiality, was obtained from all the participants; only the participants who voluntarily agreed to participate were included, and all participants were allowed to withdraw at any time without repercussions.

### 2.2. Participants

The participants consisted of 13 nurses in four geriatric hospitals located in three provinces of South Korea who had more than one year of work experience. Of the 13 participants, 4 withdrew after the first interview. Data saturation was achieved while interviewing the 9th participant. In all, 9 participants were included in the analysis. All 9 participants were female, with an average age of 36.9 years old and ranged from 28–52; over half of them had no religion (66.7%), had an associate degree in nursing (77.8%), and were staff nurses (77.8%). The average experience as a nurse was 10.8 years, ranging from 3.5 to 20 years, and experience as a nurse working in a geriatric hospital averaged 5.3 years, ranging from 3 to 10 years (Table 1).

### 2.3. Data Collection

The data were collected between August 2015 and January 2016. The participants’ facial expressions and gestures were also noted, along with the researchers’ questions and thoughts. The interviews were conducted at hospital counselling offices or at home, according to the participants’ preference, on days when they were off duty or after they had completed their shift. Individual interviews were conducted at least twice during a period ranging from one to one and a half hours. If the participants were unable to describe their experiences due to the difficulty of the research subject, the researcher would often continue the interview by asking them to think about their duties and the different emotions they experienced while performing them. The researcher kept the questions and opinions to a minimum and mainly listened to the participants’ statements, thus accepting their experiences for what they were. Moreover, researchers’ interviews with the participants were akin to everyday conversations, and the participants were asked to explain their responses again if they were unclear. The researcher encouraged the participants to continue the conversation naturally and did not make any judgments. After the interview, the researcher listened to the recordings repeatedly to transcribe them accurately. For parts requiring clarification, the researcher requested two to three additional interviews with the participants over the phone.

### 2.4. Data Analysis

Interviews were transcribed verbatim by a transcriptionist, reviewed for accuracy, and analyzed using Colaizzi’s analysis [31] and the QSR International’s NVivo-10 qualitative analysis software application. The analysis included (1) transcribing all the participants’ descriptions by reading and rereading to obtain a general sense of the whole content, (2) extracting significant statements and phrases, (3) creating formulated meanings for each significant statement and validating the meanings through discussions among researchers to reach consensus, (4) identifying and organizing formulated meanings into theme clusters and emergent themes, called categories, (5) developing an exhaustive description of themes, and (6) describing the essential structure of the experiential phenomenon. Four participants who provided abundant information were then invited to compare the researchers’ descriptive results with their experiences to verify their accuracy. The analysis was done collaboratively by all of the researchers.

### 2.5. Trustworthiness

The reliability and validity of this study were verified according to the evaluation criteria proposed by Lincoln and Guba [32]. The researcher sought to maintain confirmability, named as bracketing, to prevent their subjective opinion from having any impact by making a field note on their prejudice, assumptions, speculations, and pre-understanding. For transferability, data were collected until saturation, wherein no new statement was given by the participants. To ensure credibility, the researchers repeatedly examined the transcriptions of the raw data to find the fundamental meaning of the participants’ lived experience based on the approach of phenomenological reduction and verify whether the participants’ statements had been analyzed correctly. For this reason, the researchers showed the significant statements and the formulated meaning of the restatements to four participants to verify if they corresponded to their actual experiences. For improving dependability, five scholars with extensive experience in qualitative research were requested to evaluate the results and the overall research process. Revisions for themes, theme clusters, and categories were made.

## 3. Results

Data analysis identified three main theme categories. The theme categories abstracted were as follows: confusing values for good nursing, distress resulting from not acting the right way despite being aware, and avoiding ethical conflicts as a last resort (Table 2).

### 3.1. Confusing Values for Good Nursing

#### Uncertainty about the Right or Wrong Action for a Good Nurse: “I Don’t Know What Makes Good Nursing in the Real World”

The overall experience of confusing values for good nursing reflected the uncertainty for a good nurse about the right or wrong action. The participants were confronted with certain ethical situations where they made a choice in the face of two compelling alternatives between maintaining their values in good nursing and violating them. At the moment they found out violating their values could benefit the patients, patients’ families, or staff, they reconsidered the meaning of good nursing, agonizing about what was right or wrong.

The participants doubted whether treatment decisions by patients’ families were right. Some of the decisions seemed to benefit patients’ families at the expense of patients. Through reflecting on these cases, the participants became aware of the reasons for decisions affected by families’ financial strain, children’s burden from social pressure, and duty to support their elderly parents. The participants agonized between nursing for patients’ well-being only and nursing with consideration of practical implications. This experience was described in the following statement: “In a different way to think, I understand the son’s burden to support his sick father as a responsibility to look after old parents in Korea… Somehow, I don’t know what makes good nursing in the real world” (Participant 2).

Although the participants showed an empathetic understanding of families’ struggles, they often had no answer for how to deal with their personal discomfort toward families’ rude behaviors. They felt unpleasant; however, the question arose whether this emotion was right or wrong as good nurses. On the one hand, they believed that the purpose of nursing is to care for patients as holistic beings, and thus the families connected to the patient’s life should also be considered and supported by nurses. On the other hand, they thought nurses were also deserving of respect.

For participants who were head nurses, difficulties were encountered in balancing their roles between being a good nurse and being a good manager. As to use of restraining bands for patients’ safety, the participants’ attempts to alleviate the suffering of patients were obstructed when they recognized that it was occasionally necessary to apply a physical restraint to reduce the heavy workload of care workers such as nurse-aids. “As a nurse and as a manager for staff, I need to consider staff’s difficulties” (Participant 6).

### 3.2. Distress Resulting from Not Acting the Right Way Despite One’s Knowledge

When nurses perceived that they did not or could not act the right way despite being fully aware, they experienced distress, fear, and anger, not confusion. The fear originated from self-reflection at the prospect of violating their own nursing values. In contrast, the anger was caused by their roles as advocates being impeded by extrinsic factors, such as strained relationships between nurses and physicians, a hierarchical atmosphere in the workplace, a shortage of nurses, and incompetent and unethical substitutes for nurses, who were nursing assistants. With these experiences, they became exhausted when perceiving that they could do nothing for the patients against the hospital’s unchanging unethical attitude toward patients. Despite whistleblowing, the management of whistleblowing was unconvincing.

#### 3.2.1. Fear Originating from Seeing Oneself Violating One’s Nursing Values: “How Could I Call Myself a Nurse?”

As the participants’ experience with terminal care increased, they became more concerned with the workload rather than providing care and dignity for patients. After seeing themselves becoming desensitized to death and human indignity, the nurses had grown afraid of being accustomed to nursing with these work processes and formed habits.

When I first witnessed the passing of a patient, it was really sad. But after seeing it many times, it just feels like part of work. Now, I’m more concerned about the paperwork I have to do. Work comes first now. I get insensible to death as it happens often at geriatric hospitals. And I get afraid of myself. How could I call myself a nurse? I wasn’t like that before, what happened to me?

(Participant 3)

#### 3.2.2. Anger Resulting from Failures in Playing a Role as an Advocate: “The Most Annoying Thing Is, I Had to be Still”

For the participants, it was challenging to express their opinions on physicians’ unethical attitudes and behaviors due to unstrained relationships between nurses and physicians even though physicians were aware of nurses’ advocacy roles for their patients, which made nurses feel indignant. They believed that physicians were aware of the hierarchy between nurses and themselves; if nurses discussed physicians’ unethical behaviors with them, the responses from the physicians would have been ignorance, expressions of spite, or arguments, and regardless, physicians’ attitudes would remain fixed. For this reason, no participants had attempted to express their perspectives on physicians’ unethical behaviors. “I cannot understand why they think of my opinion as a challenge toward their authority, like how dare you! My opinion is just for the patient (in an elevated tone of voice). Doctors have so much power!” (Participant 2).

Further anger emerged as exasperation when nurses had to be silent in ignoring senior nurses’ unethical attitudes and behaviors due to their workplace atmosphere, even though they knew they had to protect the patients’ dignity and shield them from harm. The hierarchy in their workplaces was strongly fixed with no one ever overcoming it, and thus they had to accept it. No participants had discussed their senior nurses’ unethical behaviors with them against such a hierarchical climate.

The most annoying thing is, I had to be still despite realizing senior nurses’ unethical behaviors. Nobody brings up those problems in my ward for the patients. I mean, as a junior nurse, I have no choice but to have options. If I do that, I might be treated as a rude junior nurse or a freaky one… I just have to follow the group rules.

(Participant 4)

Although all participants endeavored to deliver their optimal care as much as possible, it was often not achieved due to a shortage of nurses and inadequate hospital management; that is, many hospitals hired nursing assistants to address a shortage of nurses due to the budget. This prompted nurses to feel angry because nurses could still not provide proper and safe care to their patients due to a shortage of nurses. Moreover, incompetent and unethical behaviors by numerous nursing assistants even harmed patients.

The policy allows hiring nursing assistants instead of nurses. So, which hospital would hire an expensive nurse? Of course, they would hire nursing assistants. I’m trying to care for my patients as much possible as I can though, I’m busy. But, I can’t handle even so many nursing assistants with problems. They are not capable of delivering appropriate care for my vulnerable and weak patients. In my hospital, most older patients need to be taken care of very carefully but many nursing assistants seem to have little knowledge of caring for older patients or (follow) necessary ethical rules as care workers.

(Participant 7)

#### 3.2.3. Being Exhausted by Hospitals’ Unchanging Unethical Attitude: “I Am Only One of Their Employees”

The participants were disillusioned with hospitals’ attitudes toward patient treatment only for financial benefits and fixed attitudes despite whistleblowing by employees. Aside from this, the participants realized that they could not stand for their patients’ rights to know the truth, thereby feeling frustrated and disappointed by the unconvincing way the whistleblowers were treated by their managers and hospitals.

The top priority for healthcare professionals is honesty. The patients have rights to know the truth. But, that’s the hardest part here… (exasperated sigh). The hospital and its manager never want to make a big deal out of it. I’m sick of them, they merely treat patients as money… but, it’ll be hard to continue working as a whistleblower. Anonymity is not guaranteed. No one is willing to come forward to report to the manager or management. How could I change that? I am only one of their employees.

(Participant 1)

### 3.3. Avoiding Ethical Conflicts as a Last Resort

#### Helplessly Leaving the Job when Repetitive Unmanageable Ethical Problems Occur Beyond One’s Control: “If You Can’t Stand the Heat, Get Out of the Kitchen”

All participants demonstrated their will convincingly, leaving their job rather than abandoning their ethical beliefs where the unmanageable ethical problems occurred beyond their control repeatedly. They were livid at having to leave their job due to an unchanged organization. However, the nurses could not react proactively or file external reports regarding ethical conflicts because they found themselves powerless in such a situation. They eventually suffered from helplessness. One of the participants had even left her previous job due to this reason.

Because of that hospital, I couldn’t abandon my ethical beliefs as a nurse. I’m just an employee. A regular employee cannot make changes that will happen hospital-wise. The hospital will not give up profits just because I told them what they’re doing is wrong. You know the proverb—If you can’t stand the heat, get out of the kitchen. That’s why. Many hospitals are still like that.

(Participant 6)

## 4. The Essential Structure of Ethical Conflicts by Nurses in Geriatric Hospitals in South Korea

Pervasive throughout nurses’ descriptions of their lived experiences of ethical conflicts was confusion, distress, and avoidance. Nurses were confused about whether violating their nursing values might be the right thing because it could benefit patients, families, or staff. In the process, each benefit resulting from maintaining values for good nursing and violating them was compared in practical terms, without prioritizing vital values in nursing or applying moral principles. No ethical conflicts experienced by the nurses involved confusion. When nurses perceived that they did not or could not act the right way despite being fully aware, they felt fear and anger, not confusion. The emotion of fear arose from seeing oneself changed, unknowingly violating their nursing values relating to patient care. However, they struggled to advocate for patients due to various extrinsic factors, which were related to tensions with physicians, a hierarchy in the workplace, and hiring nursing assistants whose incompetence and unethical behaviors jeopardized patients. As these experiences accumulated, nurses became exhausted when they recognized they could not advocate for patients against the hospital’s unchanging unethical attitudes and behaviors toward patients and employees. After experiencing repeated, unmanageable ethical conflicts, nurses found ethical conflicts difficult to resolve on their own, hospitals incompetent, and themselves helpless. Nurses acted on intentions to leave their job as a last resort to avoid ethical conflicts rather than compromising their beliefs in nursing. In their words, “If you can’t stand the heat, get out of the kitchen”.

## 5. Discussion

This research undertook an exploration of the meaning of the experiences of ethical conflicts by nurses in geriatric hospitals in South Korea. The major theme of the ethical conflicts was “confusing values for good nursing”. All participants, regardless of age or years of experience, often expressed uncertainty about their values of good nursing in situations where they perceived that actions based on other values could benefit patients, patients’ families, or staff. The benefit was evaluated by a simple comparison of the utility of consequences according to their ethical beliefs on nursing and other values, without prioritizing any central value in nursing such as “patient-centered care” [33], or applying principles of ethics, such as “respect for persons or autonomy” [34]. This phenomenon is presumed to be ethical conflict caused by moral uncertainty, “arising when one is unsure whether there is an ethical dilemma or not, or if one assumes there is, one is unsure what principles or values apply in the ethical conflicts” [35]. This result contrasts with previous findings that nurses experience more moral distress rather than moral uncertainty [36], which occurs among novice nurses or nursing students more frequently [37], and the finding that Finnish nurses resolve ethical conflicts pertaining to the decision of discharge from elderly care by ethical reasoning based on ethical principles, namely “ethics of care” and “justice” [26].

This difference may be attributable to the idiosyncrasies of the participants and of the workplace environments, whereby the extent of moral uncertainty can be accelerated by a lack of individual ethical knowledge and deficit or absence of ethical guidance in the hospitals. Indeed, although ethics in nursing education has been emphasized more recently, among quite a few of the South Korean nurses, ethical reasoning and decision-making were not based on ethical knowledge, but rather on conventions, expectations of others, and uncertainty [37]. Other research has stressed the enhancement of ethics in nursing education by evidencing that a lack of ethical knowledge leads to moral uncertainty [38,39]. In this vein, for the sake of improving ethical knowledge, it is suggested that focus is placed on the enhancement of ethical competence in the field of nursing. According to Gallagher, ethical competence is defined as the attainment of ethical knowledge that enables nurses to perceive and analyze a given situation accurately so that they can reflect critically on what they know, who they are, and what they do [40]. Cannaerts et al. [41] also argued that the development of ethical competence among nurses is required to respond adequately to the ethical demands of current healthcare. Further research on ethical competence in the context of nursing is required so that moral uncertainty resulting in ethical conflicts can be minimized. Another source for moral uncertainty among nurses can be the deficit or absence of ethical guidance from hospitals. Previous studies have shown that South Korean nurses experience ethical conflicts due to moral uncertainty related to obscure ethical guidelines [42]. Jang and Oh [33] emphasized the necessity for clear ethical guidance and norms through open communication between hospitals and employees for lessening moral uncertainty and emotional outrage. The establishment of clear ethical guidance or rules can alleviate anxiety about uncertainty, and thereby promote organizational commitment among nurses [43] and quality of care [44]. In consideration of the previous findings, clear ethical guidelines or regulations could alleviate a specific ethical conflict experienced by the participants in our study between personally unpleasant emotion and nurses’ roles to support patients’ families when they are faced with families’ rudeness. According to the law in South Korea, threats or verbal or physical abuse toward workers, including nurses, must be punished as written in Article 26-2 of the Occupational Safety and Health Act since 2018 [45]. Healthcare organizations are obliged to actively establish their ethical rules based on this national law and inform their employees of them. However, it should be noted that one-sided and norm-based ethical guidance not accompanied by discussion can impede appropriate nursing care and thus undermine professional autonomy [46].

It was remarkable to realize that emotional distress experienced by nurses resulted from seeing themselves becoming desensitized to death. Interestingly, the participants were aware of the right actions but nevertheless had difficulties behaving ethically as nurses facing patients’ deaths or the value of a human being’s life. They discovered themselves in such situations, thereby becoming distressed. Comparing this to the mechanism of moral distress, the consequence of both is similar in terms of psychological distress, however, their sources are clearly different. In other words, the source of moral distress is extrinsic factors (e.g., organizational constraints), while on the contrary, due to intrinsic factors (e.g., seeing oneself desensitized to death), nurses can feel distressed when they do not perform ethically right actions despite their awareness. This response to patient death was found in other research among nurses in end-of-life care settings [47,48], as well as a study on exploring reflections among medical students on dying patients [49]. According to the previous findings, when faced with patient’s death, the more medical students employed “denial” or “ignorance” as self-defense instead of sharing grief with their patients’ families, the more desensitized they became to patients’ deaths. Over time, they became anxious about their potential desensitization to death due to understanding that such attitudes were against physicians’ values of being patient-focused [49]. Even if they endeavored to find solutions to this in their hospitals, there was little systemic support to relieve their anxiety. Given this, it could be recommended that there should be place or time to fulfil an emotional or spiritual need among nurses in geriatric hospitals when facing a patient’s death.

All participants experienced ethical conflicts as “being distressed”, namely moral distress, which means not being able to do the right thing despite being aware of it [35]. In particular, failure in playing a role as an advocate resulted from unmanageable constraints that triggered nurses’ distress. This result is in line with other research on nurses in various settings [6,42]. This can be explained by the recent argument of Andrew Jameton, who first addressed the concept of moral distress among nurses by showing that nurses’ ethical concerns were heartfelt, and they contemplated the professional role of their emotion in providing compassionate care to patients, and thereby it is significant to include the emotional aspect of moral problems in the nursing context [50]. In our study, indeed, participants also passionately expressed their emotions in various ways, explaining failure in playing the role of advocate for patients. This result can account for the previous findings that the perception of advocate roles among nurses is inspired by an altruistic attitude with empathy, responsibility, and compassion toward the vulnerability of elderly patients [13,51]. Choe, Kang, and Lee [23] revealed in their qualitative research that emotional distress was one of the barriers to ethical nursing practice for older adults in long-term facilities. The sources of moral distress were perceived by nurses as unethical climate related to peers, physicians, managers, and hospitals, lack of ethical leadership, and hiring nursing assistants to solve the shortage of nurses, with nursing assistants sometimes displaying incompetence and unethical behaviors. These phenomena are consistent with previous empirical research [2,52,53] and the recent literature review regarding ethical dilemmas in nursing by Rainer, Schneider, and Lorenz [36], who found that moral distress experienced by nurses arises from ethical dilemmas due to organizational constraints such as lack of nurses’ authority in caring, regardless of culture or region.

To address this chronic problem, not only an impartial stance toward ethical problems from hospitals but also the accommodation of individual nurses’ ethical behavior based on their own ethical beliefs can be recommended. Ethical behavior, as one of the fundamental components of ethical competency [40], should be further emphasized in nursing ethics education for nurses and nursing students. Ethical behavior, namely moral behaviors motivated by moral courage, can alleviate moral distress among nurses behaving on the basis of their ethical principles and beliefs against organizational constraints [54,55]. Rushton [56], meanwhile, highlighted that moral resilience, which is defined as “the ability to respond positively to the distress and adversity caused by an ethically complex situation”, can be useful to mitigate moral distress among nurses. Further exploration of this is needed to verify the impact of ethical behavior and moral resilience on moral distress in the future.

When the participants were confronted with unmanageable ethical conflicts repeatedly, they felt hopeless and found themselves incompetent in contending with hospitals, which were indifferent toward nurses’ ethical conflicts, even worsening their experiences, thereby leaving their jobs to avoid ethical conflicts. This result is consistent with other research [57,58,59] and corresponds to the model of the moral residue crescendo, which sees unsolved moral distress generating moral residue, accumulating over time, and thereby possibly damaging one’s moral integrity; nurses could withdraw from troubling cases or leave their positions to cope with this threat [37,60]. On the other hand, in the situation of unavoidable ethical conflicts, some nurses could achieve personal development by reflecting on one’s and others’ morality and caring for patients more actively despite small numbers [53]. Further research is needed to explore which attributes make those nurses engage in such acts and to verify the effect of a supportive system or program to assist in resolving ethical conflicts.

### 5.1. Implications

This research highlighted the meaning of ethical conflicts experienced by nurses in geriatric hospitals in South Korea. The findings from this research can provide ideas and approaches for resolving ethical conflicts, thereby encouraging nurses to uphold their nursing values and urging organizations to promote ethical attitudes. For example, on the basis of the three main themes in this study, further research can be suggested to identify the levels of moral uncertainty, moral distress, and turnover due to ethical conflicts among geriatric nurses; furthermore, nurse leaders could develop nursing ethics education programs to decrease moral uncertainty or policies to alleviate moral distress by nurses. Ultimately, this research can make a contribution to the improvement of nursing environments, enabling nurses to deliver improved quality of care for the patients in geriatric hospitals.

### 5.2. Limitations

This research had some limitations. First, all of the participants were females because male nurses account for only 3% of registered nurses in South Korea [61]. Exclusive research on ethical conflicts experienced by male nurses may be conducted in the near future for comparison. Second, some of the participants decided on early withdrawal in the middle of this research because of the pressure of the hospital possibly suspecting them to be whistleblowers. Considering this, what the remaining participants expressed about their ethical conflicts might not be complete since it might have been hard for them to describe it. For instance, some issues resulting in ethical conflicts might be related to violations of law. It could be suggested that further research regarding experiences of ethical conflicts includes nurses who can freely explain their stories, such as those who have left their hospitals.

## 6. Conclusions

This research explored the meaning underlying nurses’ experiences of ethical conflicts in geriatric hospitals in South Korea. This could be understood with three phenomena: “confusion”, “distress”, and “avoidance”. For geriatric nurses to cope with ethical conflicts successfully, establishing clear ethical guidance should be made mandatory, as well as continuing ethics education that improves ethical knowledge and ethical behaviors, and a supportive system or program to resolve ethical conflicts experienced by nurses.

## Figures and Tables

**Table 1:** Characteristics of the participants.

No.	Age	Gender	Marital Status	Religion	Education *	Current Position	Years of Experience as a Nurse	Years of Experience in a Geriatric Hospital
1	32	Female	Single	No religion	ADN	Staff nurse	8	4
2	38	Female	Married	No religion	ADN	Staff nurse	4.3	3.3
3	35	Female	Married	No religion	ADN	Staff nurse	8	3
4	28	Female	Single	No religion	BSN	Staff nurse	3.5	3.5
5	33	Female	Single	Buddhist	ADN	Staff nurse	11	8
6	36	Female	Married	Christian	ADN	Head nurse	14	10
7	40	Female	Married	No religion	ADN	Staff nurse	15	4
8	38	Female	Married	No religion	ADN	Staff nurse	14	4
9	52	Female	Married	Christian	BSN	Head nurse	20	10

* ADN: Associate’s Degree in Nursing; BSN: Bachelor of Science in Nursing.

**Table 2:** Summary of themes, theme clusters, and categories.

Categories	Theme Clusters	Themes
Confusing values for good nursing	Uncertainty about the right or wrong action for a good nursing	Doubt about treatment decisions by families Uncertainty of how to deal with families’ rudeness: as a person vs. as a nurse Difficulties in balancing the roles between being a good nurse and being a good manager: to alleviate patients’ sufferings vs. solicitude for consideration of hospital staff’s workload
Distress resulting from not acting the right way despite one’s knowledge	Fear originating from seeing oneself violating one’s nursing values	Being afraid of seeing oneself become desensitized to death
	Anger resulting from failures in playing a role as an advocate	Being indignant for having difficulty in fulfilling nurses’ role and unstrained relationships between nurses and physicians Frustration at having no choice but to be silent despite observing senior nurses’ unethical attitudes and behaviors Anger at the low quality of nursing care resulting from hiring nursing assistants to solve the shortage of nurses and from nursing assistants’ incompetence and unethical behaviors
	Being exhausted by the hospital’s unchanging unethical attitude	Weariness of hospital’s attitude in referring to patients in terms of money Being frustrated because of hospital management’s way of handling whistleblowing
Avoiding ethical conflicts as last resort	Having no choice but to leave the job	Helplessly leaving the job when repetitive unmanageable ethical problems occur beyond one’s control
